# Evaluating treatment outcomes and durations among cases of smear-positive pulmonary tuberculosis in Yemen: a prospective follow-up study

**DOI:** 10.1186/s40545-017-0124-8

**Published:** 2017-12-01

**Authors:** Ammar Ali Saleh Jaber, Amer Hayat Khan, Syed Azhar Syed Sulaiman

**Affiliations:** 1 School of Pharmaceutical Sciences, Universiti Sains Malaysia, Penang, Malaysia; 2 School of Pharmacy, Lebanese International University, Taiz, Yemen

**Keywords:** Khat, Yemen, Tuberculosis, Comorbidities, United Nations Children’s emergency fund (UNESCO), Treatment duration, Treatment success

## Abstract

**Background:**

Evaluating outcomes after tuberculosis (TB) treatment can help identify the primary reasons for treatment success or failure. However, Yemen has a treatment success rate that remains below the World Health Organization’s target. This study aimed to identify factors that were associated with unsuccessful treatment and prolonged treatment (>1 year).

**Method:**

Newly diagnosed cases of smear-positive pulmonary TB were prospectively followed at two centers (Taiz and Alhodidah, Yemen) between April 2014 and March 2015. Standardized forms were used to obtain information from the patients regarding their socio-demographic and clinical characteristics, treatment duration, and TB-related information. Multivariate logistic regression analyses were performed to identify factors that were associated with unsuccessful treatment and prolonged treatment (>1 year).

**Results:**

The study included data from 273 cases of newly diagnosed TB, with treatment being successful in 227 cases (83.1%) and unsuccessful in 46 cases (16.9%). Among the 46 patients with unsuccessful treatment, 29 patients (10.6%) stopped treatment, 6 patients (2.2%) transferred to another facility, 6 patients (2.2%) experienced treatment failure, and 5 patients (1.8%) died. The multivariate logistic regression analyses revealed that unsuccessful treatment was associated with female sex, illiterate status, and the presence of comorbidities. Prolonged treatment durations were associated with living in a rural area, smoking, chewing khat, a cough that lasted for >3 weeks at the beginning of treatment, and bilateral cavities during radiography.

**Conclusion:**

These results confirm that the treatment success rate in Yemen is lower than the World Health Organization’s target for smear-positive pulmonary tuberculosis. Targeting the risk factors that we identified may help improve treatment outcomes. Furthermore, it may not be prudent to re-treat patients using first-line TB drugs after an initial treatment failure.

## Background

The World Health Organization (WHO) estimates that >30% of the global population has latent or active tuberculosis (TB), and approximately 8 million new cases are detected every year [1]. In addition, TB is considered a deadly infection that causes 1.5 million deaths per year worldwide [2–4]. Furthermore, studies from the pre-chemotherapy era had revealed that approximately 70% of people with sputum smear-positive pulmonary TB died within 10 years. Thus, evaluating treatment outcomes among patients with newly diagnosed smear-positive TB can help determine the success of national TB control programs (NTCPs) [2]. Furthermore, NTCP data can be used to identify treatment weaknesses and related risk factors, which can be targeted to improve the programs’ overall performance [5, 6].

Yemen has a relatively low treatment success rate, compared to the WHO’s target of 90% [4]. In addition, TB is the fourth leading cause of death in Yemen and is considered a major priority by the Ministry of Health [5]. Thus, prospective evaluation of treatment outcomes can be used to identify risk factors that are associated with TB treatment outcomes and predictors of TB treatment success [6]. Furthermore, the WHO recommends analyzing smear-positive and smear-negative pulmonary and extrapulmonary TB as separate cohorts, and no studies have evaluated the risk factors for unsuccessful treatment of newly diagnosed smear-positive TB in Yemen. Moreover, treatment duration is an important consideration, as a duration of >6 months can significantly affect the quality of life and clinical outcomes of patients with TB [7]. Therefore, the present study evaluated newly diagnosed cases of smear-positive TB at two centers in Yemen, and evaluated factors for associations with unsuccessful treatment outcomes and prolonged treatment durations.

## Methods

### Study setting

This prospective follow-up study was performed at two major TB centers that are located in the cities of Taiz and Alhodidah in Yemen. These centers have central laboratories for diagnosing TB (i.e., they perform sputum testing and isolation cultures), are used as both research and surveillance centers [5], and employ >60 health workers, including physicians, laboratory technicians, officers, and pharmacists.

All patients with confirmed smear-positive pulmonary TB are advised to take anti-TB drugs daily during the intensive phase of treatment and weekly during the continued phase [6]. These treatments are administered as oral tablets that contain isoniazid, rifampicin, pyrazinamide, and ethambutol, with doses that are determined based on the patient’s body weight. The intensive phase involves 2 months of treatment using isoniazid, rifampicin, pyrazinamide, and ethambutol, while the continued phase involves 4 months of treatment using only isoniazid and rifampicin [5]. Patients undergo monitoring and sputum smear testing at the end of the intensive phase, after 5 months, and during the last week of their treatment. If patients have a negative smear result at the end of the intensive phase, the treatment is continued as planned until the end of the program. If patients have a positive smear result, the treatment is extended for 1 month and the smear test is repeated at the end of the third month. If a negative smear result is subsequently observed at the end of the third month, the treatment is continued as planned. If positive results are observed after 3 months or 5 months, culturing and susceptibility testing is repeated. A positive smear result after 5 months is considered treatment failure.

In Yemen, the main tools for diagnosing smear-positive pulmonary TB are a direct sputum smear test using acid-fast bacillus microscopy, radiography, and the physician’s clinical discretion. Sputum testing is also used to evaluate the outcomes of pulmonary TB. Other centers may perform additional testing for high-risk cases, such as testing for HIV, liver function, kidney function, and fasting blood glucose.

### Study design and data collection

Between April 2014 and March 2015, 353 patients with newly diagnosed smear-positive pulmonary TB were registered in the two centers. However, we only included data from 273 patients who fulfilled the following criteria: age of ≥15 years, newly diagnosed smear-positive pulmonary TB, and willing to participate in the study [8]. Patients were excluded if they had unconfirmed TB, retreatment, multi-drug resistant TB, relapse, cancer, severe comorbid conditions (e.g., end-stage renal failure and advance heart disease), were pregnant, or were unwilling to participate. All patients were followed during their treatment until an outcome was recorded (treatment success, treatment failure, stopped treatment, transferred to another facility, or death).

A standardized data collection form was used to obtain the patients’ demographic and clinical characteristics, such as age, sex, marital status, chewing khat, smoking, side effects of treatment, comorbidities, body mass index, residential area, level of education, laboratory results, clinical findings, and treatment outcomes. The participants also completed a questionnaire at their diagnosis regarding their TB-related knowledge and experiences. A medical chart and standardized form for all TB patients were created at their diagnosis and maintained until the end of their treatment period. A few sociodemographic factors were obtained directly from the patients, such as BCG test results, family history of TB, income, and level of education [3, 8].

The questionnaires contained 6 knowledge-related questions, 2 stigma-related questions, and 2 treatment-related questions. The stigma questions were scored on a scale of 0–2, with single-question scores of 1–2 being considered indicative of stigmatization. The knowledge questions were scored on a scale of 1–3, with good scores being defined as a total score of 4–6 and poor scores being defined as a total score of 1–3 [9]. The original questionnaire was sent to four international TB experts, as well as NTCP physicians in Yemen, and their comments were used to create the final version of the questionnaire. A pilot study was performed to evaluate the questionnaire’s reliability and validity, and 30 patients with TB completed the questionnaires at the participating centers after providing informed consent. The patients were asked to evaluate the questionnaire’s simplicity, and their comments were also considered when creating the final version. Reliability testing revealed that the questionnaire had a Cronbach’s alpha value of 0.7. The final questionnaire was translated from Arabic into English for evaluation, and then back-translated into Arabic at Sana’a University (an English-language university). These translations were validated at the UNESCO office in Sana’a and by language experts from Sana’a University. In addition, approval for the study’s protocol was obtained from the Ministry of Public Health in Yemen and the national tuberculosis control program.

Six trained pharmacists and nurses (three in each city) collected the data from the patients’ TB cards and medical records. Treatment outcomes were reported based on the 6-month target from the WHO guidelines [10].

### Definitions of terms

#### New case

Patients who had never received treatment for TB or who had received anti-TB drugs for <1 month.

#### Successfully treated

Patients who completed treatment or who were cured.

#### Stigma

An undesirable or discrediting attribute that an individual possesses, which reduces their social status.

#### Khat

An evergreen shrub of the Celastraceous family (*Catha edulis*) that grows in parts of the Middle East and eastern Africa. Its leaves are chewed for their stimulating effect [11].

#### Prolonged duration

A course of treatment that was >6 months.

### Statistical analysis

All data were analyzed using PASW software (version 22; IBM Corp, Armonk, NY). Categorical variables were reported as number and percentage, and TB treatment outcomes were categorized as successful outcomes (cured or completed treatment) or unsuccessful outcomes (treatment failure, stopped treatment, transferred to another facility, and death) [3]. Univariate logistic regression analyses were used to evaluate the associations of the patients’ characteristics with unsuccessful treatment outcomes and prolonged treatment durations. Multivariate logistic regression analyses were used to evaluate the characteristics’ independent associations with these outcomes. The results were reported as odds ratios (OR), 95% confidence intervals (CIs), and standard error. Differences with a *p*-value of <0.05 were considered statistically significant.

### Ethical considerations

All participants provided written informed consent before being included in this study. Ethical approval for this study was obtained from the National Committee of Health (Ministry of Health-Sana’a/Yemen) and the NTCP, which issued a collaboration letter to the two participating TB centers. All patient data were anonymized prior to the analyses.

## Results

A total of 353 patients with smear-positive pulmonary TB were registered in the cities of Taiz and Alhodidah, although only 273 patients fulfilled the inclusion criteria and their demographic characteristics are shown in Table 1. Most patients were male (54.9%), 40.3% were 16–25 years old, 77.7% were of reproductive age (<45 years old), and 52.7% had a body mass index (BMI) of <18.5 kg/m^2^. Although the study was performed in urban areas, approximately 31.5% of the patients came from rural areas and 67% were living under the poverty line (monthly income: <$50 dollars or <10,000 rial). Moreover, 55.3% of the patients were married and 58.6% were unemployed. Illiteracy was common (44.7%), and most illiterate patients were from Al Hudaydah (56.6%). Smoking was common (70%, with 71.8% of smokers smoking <20 cigarettes/day), and 64.5% of the patients chewed khat. We observed that 32.6% of the patients had a family history of TB, and that 18.3% underwent BCG vaccination. Comorbid conditions were observed in 161 patients.

**Table 1:** Socio-demographic characteristics of 273 smear-positive tuberculosis cases that were registered between April 2014 and March 2015

	City		Patients with tuberculosis n (%)
	Taiz, n (%)	Alhodidah, n (%)	
Sex			
Male	72 (48)	78 (52)	150 (54.9)
Female	73 (59.3)	50 (40.7)	123 (45.1)
Age (years)			
16–25	69 (62.7)	41 (37.3)	110 (40.3)
26–35	37 (52.9)	33 (47.1)	70 (25.6)
36–45	18 (56.3)	14 (43.8)	32 (11.7)
46–55	13 (40.6)	19 (59.4)	32 (11.7)
56–65	4 (22.2)	14 (43.8)	18 (6.6)
≥ 66	4 (36.4)	7 (63.3)	11 (4)
Weight (kg)			
≤ 30	2 (18.2)	9 (81.8)	11 (4)
31–40	43 (61.4)	27 (38.6)	70 (25.6)
41–50	69 (52.7)	62 (47.3)	131 (48)
51–60	19 (47.5)	21 (52.5)	40 (14.7)
61–70	8 (53.3)	7 (46.7)	15 (5.5)
> 70	4 (66.7)	2 (33.3)	6 (2.2)
BMI (kg/m^2^)			
< 18.5	80 (55.6)	64 (44.4)	144 (52.7)
≥ 18.5	65 (50.4)	64 (49.6)	129 (47.3)
Area			
Urban	91 (48.7)	96 (51.3)	187 (68.5)
Rural	54 (62.8)	32 (37.2)	86 (31.5)
Level of education			
Illiterate	53 (43.4)	69 (56.6)	122 (44.7)
Primary	34 (54.8)	28 (45.2)	62 (22.7)
Pre-secondary	23 (82.1)	5 (17.9)	28 (10.3)
Secondary	25 (53.2)	22 (46.8)	47 (17.2)
University	10 (71.4)	4 (28.6)	14 (5.1)
Marital status			
Single	49 (45.8)	58 (54.2)	107 (39.2)
Divorced	2 (50)	2 (50)	4 (1.5)
Widow	6 (54.5)	5 (45.5)	11 (4)
Married	88 (58.3)	63 (41.7)	151 (55.3)
Employment status			
Employed	57 (50.4)	56 (49.6)	113 (41.4)
Unemployed	88 (55)	72 (45)	160 (58.6)
Smoking habit			
Yes	110 (57.6)	81 (42.4)	191 (70)
No	35 (42.7)	47 (57.3)	82 (30)
Chewing khat ^a^			
Yes	90 (51.1)	86 (48.9)	176 (64.5)
No	55 (56.7)	42 (43.3)	97 (35)
Monthly income (rial^b^)			
≤ 10,000	98 (53.6)	85 (46.4)	183 (67)
> 10,000	47 (52.2)	43 (47.8)	90 (33)
Stigma			
Yes	49 (41.2)	70 (58.8)	119 (43.6)
No	96 (62.3)	58 (54.7)	154 (56.4)
Knowledge			
Poor (1–3)	23 (59)	16 (41)	39 (14.3)
Good (4–6)	122 (52.1)	112 (47.9)	234 (85.7)
Family history of tuberculosis			
Yes	61 (68.5)	28 (31.5)	89 (32.6)
No	84 (45.7)	100 (54.3)	184 (67.4)
BCG vaccination			
Yes	42 (84)	8 (16)	50 (18.3)
No	103 (46.2)	120 (53.8)	223 (81.7)

*BMI* body mass index, *BCG* Bacillus Calmette–Guerin

^a^khat: a shrub that grows in parts of East Africa and Yemen, ^b^ rial: one dollar is approximately equivalent to 215 rial

Table 2 shows the patients’ clinical characteristics. Approximately 47.3% of the patients had ≥5 clinical symptoms at the start of treatment, and 83.5% had a > 3-week history of coughing. Chest radiography revealed that unilateral lesions in 61.2% of the patients and single-lung cavities in 41.8% of the patients. The outcomes were cure for 186 patients (68.1%), completed treatment for 41 patients (15%), treatment failure for 6 patients (2.2%), stopped treatment for 6 patients (2.2%), transfer to another facility for 29 patients (10.6%), and death for 5 patients (1.8%) **(**Table 3).

**Table 2:** Clinical characteristics of the 273 patients with smear-positive tuberculosis

	City		Patients with tuberculosis, n (%)
	Taiz, n (%)	Alhodidah, n (%)	
Number of clinical symptoms			
≤ 2	29 (53.7)	25 (46.3)	54 (19.8)
3–4	50 (55.6)	40 (44.4)	90 (33)
≥ 5	66 (51.2)	63 (48.8)	129 (47.3)
Cough history			
< 3 weeks	37 (82.2)	8 (17.8)	45 (16.2)
≥ 3 weeks	108 (47.4)	120 (52.6)	228 (83.5)
Radiography lesions			
Unilateral	92 (55.1)	75 (44.9)	167 (61.2)
Bilateral	52 (53.1)	46 (46.9)	98 (35.9)
No test performed	1 (12.5)	7 (87.5)	8 (2.9)
Lung cavitation			
No cavities	42 (72.4)	16 (27.6)	58 (21.2)
One	57 (50)	57 (50)	114 (41.8)
Two	34 (48.6)	36 (51.4)	70 (25.6)
Three or more	11 (47.8)	12 (52.2)	23 (8.4)
No test performed	1 (12.5)	7 (87.5)	8 (2.9)
Comorbidity			
Yes	19 (43.2)	25 (56.8)	44 (16.1)
No	126 (55)	103 (45)	229 (83.9)

**Table 3:** Treatment outcomes for patients with prolonged tuberculosis based on the WHO/IUALTLD criteria

Treatment outcome	Patients, n (%)	Groups, n (%)
Successful		
Cure	186 (68.1)	227 (83.1)
Completed treatment	41 (15.0)	
Unsuccessful		
Treatment failure	6 (2.2)	46 (16.9)
Died	5 (1.8)	
Stopped treatment	29 (10.6)	
Transferred to another facility	6 (2.2)	
Total	273 (100)	273 (100)

### Risk factors associated with unsuccessful treatment outcomes

The results of the univariate analyses revealed that female sex, illiterate status, unemployment, smoking, an income of <10,000 rials/month, and comorbidities were associated with unsuccessful treatment outcomes. The significant univariate factors were entered into the multivariate analyses, which revealed that female sex, illiterate status, and comorbidities were independently associated with unsuccessful treatment outcomes (Table 4).

**Table 4:** Independent risk factors for unsuccessful treatment outcomes

		Treatment outcome n (%)	Univariate OR (95% CI)	Multivariate AOR (95% CI)	
		Successful	Unsuccessful		
Sex	Male	114 (76)	36 (24)	Ref	
	Female	113 (91.9)	10 (8.1)	0.470 (0.281–0.787)	0.248 (0.239–0.874)
Age, years	≤ 45	177 (83.5)	35 (16.5)	Ref	
	>45	50 (82)	11 (18)	1.210 (0.678–2.161)	
Area	Urban	152 (81.3)	35 (18.7)	Ref	
	Rural	75 (87.2)	11 (12.8)	1.054 (0.630–1.763)	
Education level	Literate	127 (82.5)	27 (17.5)	Ref	
	Illiterate	100 (84)	19 (16)	2.155 (1.22–3.801)	2.294 (1.272–4.137)
Occupation	Employed	90 (79.6)	23 (20.4)	Ref	
	Unemployed	137 (85.6)	23 (14.4)	1.591 (0.982–2.575)	0.980 (0.379–2.573)
BCG vaccination	Yes	46 (92)	4 (8)	Ref	
	No	181 (81.2)	42 (18.8)	1.651 (0.849–3.211)	
Marital status	Married	128 (84.8)	23 (15.2)	Ref	
	Unmarried	99 (81.1)	23 (18.9)	0.822 (0.506–1.334)	
Smoking habit	No	164 (85.9)	27 (14.1)	Ref	
	Yes	63 (76.8)	19 (23.2)	1.570 (0.943–2.613)	1.474 (0.867–2.578)
Chewing khat ^a^	No	84 (86.6)	13 (13.4)	Ref	
	Yes	143 (81.3)	33 (18.8)	1.397 (0.840–2.322)	
Income (rial^b^)	>10,000	73 (81.1)	17 (18.9)	Ref	
	≤10,000	154 (84.2)	29 (15.8)	0.651 (0.397–1.068)	
Stigma	No	127 (82.5)	27 (17.5)	Ref	
	Yes	100 (84)	19 (16)	0.683 (0.413–1.131)	
Knowledge	Good (4–6)	193 (82.5)	41 (17.5)	Ref	
	Poor (1–3)	34 (87.2)	5 (12.8)	0.831 (0.401–1.725)	
BMI, kg/m^2^	<18.5	120 (83.3)	24 (16.7)	Ref	
	≥18.5	107 (82.9)	22 (17.1)	1.043 (0.646–1.685)	
Comorbid	No	197 (86)	32 (14)	Ref	
	Yes	30 (68.2)	14 (31.8)	1.730 (0.981–3.044)	1.995 (1.108–3.590)
Family history of tuberculosis	No	150 (81.5)	34 (18.5)	Ref	
	Yes	77 (86.5)	12 (13.5)	0.890 (0.525–1.509)	

*Ref* reference group, *BMI* body mass index, *BCG* Bacillus Calmette–Guerin, *AOR* adjusted odds ratio, *CI* confidence interval

^a^khat: a shrub that grows in parts of East Africa and Yemen, ^b^ rial: one dollar is approximately equivalent to 215 rial

### Risk factors associated with prolonged treatment duration

A total of 200 patients had an intensive phase that lasted for >2 months and 78 patients had a continued phase that lasted for >4 months. Among the 273 patients, 266 patients completed the intensive phase and 227 patients completed the continued phase (Table 5).

**Table 5:** Treatment durations for cases of smear-positive pulmonary tuberculosis

Duration of treatment	Patients, n (%)
End of the intensive phase	
≤ 2 months	66 (24.2)
> 2 months	200 (73.2)
Total	266 (100)
End of the continuation phase	
≤ 4 months	149 (56)
> 4 months	78 (29.3)
Total	227 (100)

The univariate risk factors for prolonged treatment duration were entered into the multivariate analyses. These analyses revealed that prolonged treatment duration was independently associated with living in a rural area (adjusted odds ratio [AOR]: 2.358, 95% CI: 0.658–4.00), smoking (AOR: 0.054, 95% CI: 0.987–4.112), chewing khat (AOR: 2.615, 95% CI: 1.201–5.691), having a cough for >3 weeks at the start of treatment (AOR: 2.672, 95% CI: 1.053–6.782), and bilateral lesions during radiography (AOR: 2.134, 95% CI: 1.147–3.972) (Table 6).

**Table 6:** Independent risk factors for prolonged treatment duration

		Treatment duration		Univariate OR (95% CI)	Multivariate AOR (95% CI)
		≤6 months	>6 months		
Sex	Male	34 (29.8)	80 (70.2)	Ref	
	Female	23 (20.4)	90 (79.6)	0.539 (0.309–0.940)	1.623 (0.658–4.002)
Age, years	≤ 45	44 (24.9)	133 (75.1)	Ref	
	>45	13 (26)	37 (74)	0.873 (0.447–1.705)	
Area	Urban	38 (25)	114 (75)	Ref	
	Rural	19 (25.3)	56 (74.7)	2.636 (1.48–4.695)	2.358 (1.250–4.433)
Education level	Literate	34 (26.8)	93 (93.2)	Ref	
	Illiterate	23 (23)	77 (77)	0.972 (0.560–1.688)	
Occupation	Employed	25 (27.8)	65 (72.2)	Ref	
	Unemployed	32 (23.4)	105 (76.6)	0.442 (0.252–0.775)	0.633 (0.176–2.282)
BCG	Yes	12 (26.1)	34 (73.9)	Ref	
	No	45 (24.9)	136 (75.1)	1.103 (0.554–2.196)	
Marital status	Married	28 (21.9)	100 (78.1)	Ref	
	Unmarried	29 (29.3)	70 (70.7)	0.786 (0.450–1.370)	
Smoking habit	No	44 (26.8)	120 (73.2)	Ref	
	Yes	13 (20.6)	50 (79.4)	2.648 (1.453–4.82)	2.015 (0.987–4.114)
Chewing khat ^a^	No	20 (23.8)	64 (76.2)	Ref	
	Yes	37 (25.9)	106 (74.1)	3.253 (1.720–6.152)	2.615 (1.201–5.691)
Monthly income (rial^b^)	>10,000	19 (26)	54 (74)	Ref	
	≤10,000	38 (24.7)	116 (75.3)	0.421 (0.236–0.750)	0.744 (0.231–2.404)
Knowledge	Good	51 (26.4)	142 (73.6)	Ref	
	Poor	6 (17.6)	28 (82.4)	1.411 (0.669–2.975)	
Stigma	No	31 (24.4)	96 (75.6)	Ref	
	Yes	26 (26)	74 (74)	1.232 (0.710–2.136)	
Comorbidities	No	50 (25.4)	147 (74.6)	Ref	
	Yes	7 (23.2)	23 (76.7)	1.323 (0.602–2.910)	
Family history of TB	No	42 (28)	108 (72)	Ref	
	Yes	15 (19.5)	62 (80.5)	0.297 (0.667–1.214)	
Number of TB symptoms	3–4	3 (40)	7 (70)	Ref	
	≥5	54 (24.9)	163 (75.1)	2.156 (0.447–10.41)	
History of cough	< 3 weeks	12 (30)	28 (70)	Ref	
	≥3 weeks	45 (24.1)	142 (75.9)	2.393 (1.04–5.485)	2.672 (1.053–6.782)
Radiography lesions	Unilateral	41 (28.5)	103 (71.5)	Ref	
	Bilateral	16 (19.3)	67 (80.7)	2.021 (1.149–3.553)	2.134 (1.147–3.971
Lung cavitation	No	0 (0)	50 (100)	Ref	
	Yes	57 (32.2)	120 (67.8)	1.652 (0.819–3.333)	
BMI, kg/m^2^	<18.5	38 (31.7)	88 (82.2)	Ref	
	≥18.5	19 (17.8)	88 (82.2)	1.345 (0.775–2.337)	

*Ref* reference group, *BMI* body mass index, *BCG* Bacillus Calmette–Guerin, *AOR* adjusted odds ratio, *CI* confidence interval

^a^khat: a shrub that grows in parts of East Africa and Yemen, ^b^ rial: one dollar is approximately equivalent to 215 rial

## Discussion

Our study obtained the risk factors associated with unsuccessful outcome and prolongs treatment duration for all smear positive tuberculosis patients. The present study had a relatively low inclusion rate (77% of potentially eligible individuals), which is likely related to several factors. First, approximately 6% of the individuals were excluded because they did not fulfill the inclusion criteria, and 17% of the individuals refused to participate despite being otherwise eligible. A similar WHO study in Yemen has also revealed unwillingness to participate in research among patients with TB [9]. Furthermore, the present study revealed a treatment success rate of 83%, and the latest WHO report has also detailed success rates of <85% among cases of smear-positive pulmonary TB [1]. Other developing countries also have success rates that are below the WHO target, such as India, Malaysia, Ethiopia, Pakistan, and Nigeria [12–17]. The low success rate in the present study may be related to the proportion of patients who did not complete treatment (10.6%), as most patients who experienced unsuccessful treatment outcomes were from poor areas and had an income of <10,000 rial/month, which could interfere with their ability to complete treatment. For example, a South African study revealed that difficulties in travelling from villages to cities could significantly affect the ability of patients with TB to complete their treatment [18]. Another study revealed that 29% of patients with TB only receive intermittent treatment doses from clinical centers [19]. In addition, health services are difficult to access in rural areas, and 31.5% of the participants were from rural areas and would have encountered difficulty in travelling to urban TB centers for treatment. A WHO study in Yemen [20] revealed that only 30–32% of patients with TB have access to nearby health services, and >50% of patients must travel for >1 h to the nearest TB center. Moreover, failure to complete treatment could be related to low satisfaction with the provided healthcare, which is commonly observed among patients during TB treatment [8]. Therefore, improving health services in rural areas, and decreasing the travel-related burden borne by patients with TB, may help reduce the rate of patients who fail to complete treatment.

The present study revealed a treatment failure rate of 2.2%, although the latest WHO report revealed zero cases of treatment failure in Yemen [1]. Similar studies have revealed failure rates of 2.9% and 5% in Ethiopia and Nigeria, respectively [21, 22], although a different study revealed that no patients were lost to follow-up during TB treatment [12]. In this context, patients with smear-positive pulmonary TB are defined as experiencing treatment failure if a positive smear result is detected after 2 months or 5 months of treatment [5]. Similar studies in Hamburg and south India have revealed treatment failure rates of 2.3% and 2%, respectively [13, 23], while higher values have been reported in Russia and Turkey (8%) [24, 25] and lower values have been reported in Ethiopia (0.4%) and Switzerland (0.8%) [26, 27]. Interestingly, we observed that patients who experienced treatment failure had been re-treated using the same first-line anti-TB drugs, which could worsen their health and outcomes, as well as lead to drug resistance [2]. Moreover, we observed that approximately 2.2% of the participants were transferred to other TB centers, and these individuals were generally from rural areas. Thus, the economic costs (e.g., transportation, time off work) may have led patients to seek treatment closer to their homes, rather than in urban centers [17]. A recent study in Yemen revealed that 47% of patients who received treatment for TB in Sana’a were actually from rural areas [17], and similar transfer rates have been observed in Nigeria (3%) and Russia (1%) [24, 28]. A lower transfer rate has been reported in Taiwan (0.2%) [24].

The present study revealed a relatively low mortality rate (1.8%), although it is possible that this rate is underestimated based on patients who were lost to follow-up. For example, a European systematic review has indicated that proper follow-up of patients who fail to complete treatment may reveal a larger proportion of patients who have died despite receiving treatment for TB [25]. The present study also revealed a cure rate of approximately 68%, based on patients who completed treatment and had at least two negative smear results. Nevertheless, it may be difficult for the patient to produce sputum after treatment [3], and it is possible that our cure rate was overestimated, as the participating centers did not have access to advanced sputum collection methods (e.g., an ultrasonic nebulizer, gastric aspiration, or bronchoalveolar lavage) [6, 29].

### Risk factors associated with unsuccessful treatment outcomes

The multiple logistic regression analyses revealed that unsuccessful treatment outcomes were independently associated with male sex, illiterate status, and comorbidities. Illiteracy was associated with a 2.3-fold higher risk of unsuccessful treatment, and approximately 41.2% of the participants were considered illiterate. A national report regarding education in Yemen also revealed that approximately 45% of the population is illiterate, and that most illiterate individuals are women [27]. Another study of adults in Yemen (2008–2012) revealed that the literacy rate was 65% [30], and those findings are generally in agreement with our findings. Illiteracy may be an important risk factor for unsuccessful treatment, as individuals could be less aware of TB and its routes of transmission [28]. For example, a prospective study in Yemen revealed that illiterate individuals had a 3-fold higher risk of developing TB, compared to literate individuals [31]. Other studies in India [32] and Rajasthan [28] have revealed large proportions of individuals who were illiterate and who had an elevated risk of poor treatment outcomes.

Comorbidities were associated with a 1.9-fold higher risk of unsuccessful treatment, and this result highlights the need to identify, monitor, and address comorbidities among patients who are receiving TB treatment [33]. For example, comorbidities can affect treatment outcomes by influencing the patient’s weight [34] and increasing the risk of death. Our results indicate that 18.9% of the participants had comorbidities, and it is possible that routine health checkups might have identified further comorbidities. Thus, we recommend frequent monitoring for comorbid conditions.

Male sex was also a risk factor for unsuccessful treatment outcome, and similar results were observed in studies that were performed in Malaysia, Finland, Nigeria, Kenya, and Saudi Arabia [3, 29, 35–37]. Men in Yemen frequently have high-risk behaviors, such as chewing khat and smoking, which may also affect their treatment outcomes [3, 8].

### Risk factors associated with prolonged treatment duration

Prolonged treatment duration was associated with living in a rural area, smoking, chewing khat, bilateral cavities during radiography, and a cough that lasted for >3 weeks at the start of treatment. It is important to identify risk factors for prolonged treatment duration, as it can lead to drug resistance and multidrug-resistant TB [38]. In the present study, bilateral cavities were associated with a 2.13-fold higher risk of prolonged treatment (vs. unilateral cavities), and previous studies have also revealed an association between lung cavities and prolonged treatment duration, based on the increased sputum conversion rate. Patients with TB who smoke have increased global rates of morbidity and mortality [39], as well as a 70% greater risk of unsuccessful treatment outcomes, compared to nonsmokers [39]. Furthermore, previous studies have revealed that prolonged treatment duration is associated with smoking habits [40], which may be related to the lower likelihood of smear conversion during the treatment [41]. Moreover, smoking destroys the lungs and weakens the immune system, which can lead to a prolonged treatment duration [41], as well as an increased risk of developing pulmonary TB.

The present study also revealed that chewing khat was associated with prolonged treatment duration (AOR: 2.7). This may be related to the amphetamine and cathinone in khat suppressing the immune system, which could increase the likelihood of TB and prolonged treatment [42, 43]. Further studies are needed to evaluate the effects of khat on the clinical outcomes of TB treatment.

The present study also revealed that living in a rural area was associated with prolonged treatment (AOR: 2.3), which may be related to the greater transportation costs and unavailability of TB treatment in rural areas [10]. It is also possible that living in a rural area may delay the diagnosis of TB [44], which could increase the risk of prolonged treatment. For example, 83% of our participants had a cough at the start of their treatment, and patients with a cough that lasted for >3 weeks had an increased risk of prolonged treatment. A previous study has also revealed that having a cough for >3 weeks was associated with prolonged treatment during the intensive phase of treatment [45].

### Study limitations

The present study has two important limitations. First, we only evaluated cases of smear-positive pulmonary TB, and further studies are needed to explore the risk factors for all forms of TB. Second, we only evaluated centers in two cities, based on the WHO’s recommendation to evaluate cohorts at the district, city, or state level [2], and further studies are needed to evaluate patients with TB in all cities of Yemen.

## Conclusion

The present study revealed a treatment success rate in Yemen that was below the WHO’s target. Failure to complete treatment may be responsible for the low success rate, and it would be prudent to address factors that affect patients’ ability to complete their treatment. Moreover, we conclude that monitoring and addressing the risk factors that were associated with treatment outcomes and duration may help improve the likelihood of achieving favorable outcomes among cases of smear-positive pulmonary TB in Yemen.
